# Ectopic Cushing syndrome in Colombia

**DOI:** 10.20945/2359-3997000000271

**Published:** 2020-06-19

**Authors:** Vanessa Lopez-Montoya, Johnayro Gutierrez-Restrepo, Jose Luis Torres Grajales, Natalia Aristizabal, Doly Pantoja, Alejandro Roman-Gonzalez, Camilo Jimenez

**Affiliations:** 1 Facultad de Medicina Universidad de Antioquia Medellín Colombia Facultad de Medicina, Universidad de Antioquia, Medellín, Colombia; 2 Departamento de Endocrinología Universidad de Antioquia Medellín Colombia Departamento de Endocrinología, Universidad de Antioquia, Medellín, Colombia; 3 Clínica Las Américas Departamento de Endocrinología Universidad Pontificia Bolivariana Medellín Colombia Clínica Las Américas, Departamento de Endocrinología, Universidad Pontificia Bolivariana, Medellín, Colombia; 4 Universidad de Nariño Pasto Colombia Universidad de Nariño, Pasto, Colombia; 5 Departamento de Endocrinología Hospital Universitario San Vicente Fundación Medellín Colombia Departamento de Endocrinología, Hospital Universitario San Vicente Fundación, Medellín, Colombia; 6 Departamento de Neoplasia Endocrina y Trastornos Hormonales Universidad de Texas MD Anderson Cancer Center Houston Texas United States Departamento de Neoplasia Endocrina y Trastornos Hormonales, Universidad de Texas, MD Anderson Cancer Center, Houston Texas

**Keywords:** Cushing syndrome, neuroendocrine tumors, carcinoids, ectopic ACTH production syndrome

## Abstract

**Objective:**

The aim was to describe the clinical features of patients with ectopic Cushing syndrome (ECS) from Colombia and compare these findings with other series to provide the best management for these patients.

**Materials and methods:**

Records of patients with ECS from 1986 to 2017 were retrospectively reviewed; patients with a diagnosis of adrenal or pituitary Cushing syndrome (CS) were excluded.

**Results:**

Fourteen patients with ECS were analyzed in this study. The mean age was 54.4 (SD 17.1) years, and the female to male ratio was 1.33:1. Regarding the etiology of ECS, four patients had lung carcinoids (28.6%), three had small-cell lung carcinoma (21.4%), three had pancreatic neuroendocrine tumors (21.4%), one had medullary thyroid cancer (7.1%), one had non-metastatic pheochromocytoma (7.1%), one had metastatic thymoma (7.1%) and one patient had an occult source of ACTH (7.1%). The most common clinical features at presentation were moon-face, muscle weakness, diabetes mellitus and hypertension. Hyperpigmentation was present in 36% of patients, and 12 patients had hypokalemia with a mean value of 2.3 mEq/L (SD 0.71). The median basal cortisol, 24-hour urinary free cortisol (UFC) and ACTH were 30.5 ug/dL (IQR 21-59 ug/dL), 2,600 ug/24 h (IQR 253-6,487 ug/24 h) and 91 pg/mL (IQR 31.9-141.9), respectively. Thirteen patients (92.8%) had the site of the primary lesion identified. Six patients had undergone a surgical intervention to address the primary tumor. Resection was curative in 28.5% of patients. Death occurred in 57.1% of patients, and the median overall survival was 27 months. Intrathoracic tumors had the most aggressive behavior.

**Conclusions:**

ECS is a rare disease; however, it is associated with high morbidity and mortality. A rapid intervention supported by an interdisciplinary group is required to improve overall survival and quality of life

## INTRODUCTION

Ectopic Cushing syndrome (ECS) is the least frequent etiology of all Cushing syndrome (CS) types (5%-15%) ( 1 ). ECS is caused by the excessive synthesis and secretion of adrenocorticotropic hormone (ACTH) and/or corticotropin releasing hormone (CRH) from tumor cells, usually of neuroendocrine origin ( 2 ). Some cases may present symptoms of hypercortisolism prior to the tumor manifestations, however, this is not a common phenomenon ( 3 ). The prevalence of Cushing syndrome in Colombia is unknown and this is one of the few studies of this disease in this country. There is no clear characterization of ECS in low- or medium-income countries such as Colombia. As such, clinicians from these areas must extrapolate from data presented by developed countries, which have a different prevalence of chronic and infectious diseases as well as social conditions. Limited data from similar countries have been presented before ( 4 - 8 ), however, the epidemiology of countries such as Mexico may differ from that of countries close to the equatorial belt ( 9 ). Our main interest is to describe the clinical features of patients with ECS from Colombia and their resources in the healthcare system and compare these findings with other series to provide the best management for these patients.

## MATERIALS AND METHODS

### Study design and population

This is a retrospective study. We created a database with information derived from medical records from 4 different referral centers. The search was made in a database of interconsultations and hospitalization of adult endocrinology with new and known diagnosis. In the 4 hospitals, endocrinology was the only specialty responsible for patients with this diagnosis. The database had multiple variables including clinical manifestations, biochemical profiles, imaging studies, treatments, and outcomes. The information was analyzed in a Webforma. Patients with adrenal or pituitary CS were excluded.

### Statistical analysis

Statistical analyses were performed using SPSS v. 22.0 (IBM SPSS Statistics 25 ). Continuous variables are presented as the medians and interquartile ranges (IQR) or as the means and standard deviation (SD) depending on whether the distribution of the variables was normal. Categorical variables are presented as frequencies and proportions. Survival was evaluated by Kaplan-Meier analysis.

### Ethical aspects

This study was reviewed and approved by the Institutional Review Board of the participating hospitals.

## RESULTS

### Epidemiological characteristics

We found 14 cases of ECS; 8 were female and 6 were male. The female-to-male ratio was 1.33:1.0. Tables 1 and 2 present the characteristics of the patients in our cohort. The mean age was 54.4 years (SD 17.1). Most of the primary tumors were located in the thorax (57.1%): four tumors were lung carcinoids, three were small cell lung carcinomas (SCLC) and one patient had a metastatic thymoma. There were four intra-abdominal tumors (28.5%): three were pancreatic neuroendocrine tumors (pNET) and one patient had a pheochromocytoma. One patient had an advanced medullary thyroid carcinoma (9.1%). Only one case had an occult tumor. The median time of symptoms to diagnosis was 6 months (IQR: 2.25- 24) based on the patient’s description ( 3 , 10 - 15 ).

**Table 1:** Anatomical localization of tumors causing ectopic Cushing syndrome

Location	Number of patients	Percentage (%)
Thorax	8	57.2
Abdomen	4	28.6
Neck	1	7.1
Unknown	1	7.1

**Table 2:** Distribution of symptoms, signs and comorbidities in ectopic Cushing syndrome

Signs and symptoms	Comorbidities	Number of patients	Percentage (%)
Proximal myopathy		11	79
Moon face		9	64
Central obesity		8	57
Ecchymosis		7	50
Hyperpigmentation		5	36
Buffalo hump		4	29
Supraclavicular fat pads		4	29
	Arterial hypertension	13	93
	Diabetes Mellitus II	12	86
	Dyslipidemia	4	29
	Osteoporosis**	4	29
	Depression	4	29
	Congestive heart failure	3	21
	Venous thrombosis	3	21
	Amenorrhea*	1	33

* There are 8 women, but only 3 premenopausal women.

** Osteoporosis (Number of patients with densitometry).

### Clinical manifestations and comorbidities

**Figure 2:**
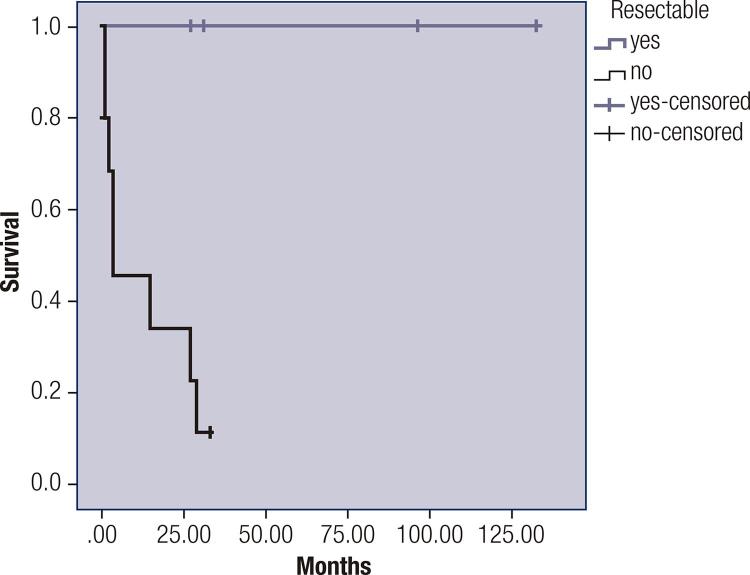
Survival according to tumor resectability. Mortality in unresectable tumors was 85%. (resectable: median not achieved, unresectable median 3 months, 95% CI 0.0-13.4) Log Rank test p = 0.013).

Figure 2 presents the clinical manifestations of the patients. Only 36% had hyperpigmentation. The associated comorbidities were hypertension in 93% of patients and diabetes mellitus in 86% of patients. Three cases (21.4%) had congestive heart failure and three (21.4%) has venous thrombotic events. One patient had nephrolithiasis without obstruction. Several cases (35.7%) with associated infectious diseases were found: malaria (Case No 1), two esophageal candidiasis (Cases No 3 and 5); urinary infection, infection by herpes zoster in dermatome T4, and periorbital cellulitis, endophthalmitis and bacteremia by methicillin-sensitive *S. aureus* (Case No 10). Two patients had septic shock associated with febrile neutropenia after receiving the first cycle of chemotherapy (Cases No 5 and 11). Four patients had osteoporosis by a confirmatory bone densitometry (28.5%). One (7.1%) had lumbar vertebral fractures not associated with metastatic disease.

### Biochemical evaluation

Table 3 summarizes the biochemical profiles of the patients. The median basal cortisol was 30.53 ug/dL (IQR 21- 59 ug/dL); UFC levels were available in twelve patients and the median was 2,600 ug/24 h, (IQR 253- 6487 ug/24 h). Four (28.5%) patients had normal UFC. These patients had lung carcinoid, pheochromocytoma, small-cell lung carcinoma, and pancreatic neuroendocrine tumor Results from high-dose dexamethasone suppression tests (HDDST) were available for 9 patients. Cortisol levels in two (14.2%) patients after HDDST revealed a suppression greater than 50% from baseline values. In the other cases, suppression was inferior. The median basal ACTH were 91 pg/ml (IQR 31.9-141.9). In three patients (Cases No 1, 4 and 14), the values were above 200 pg/mL, however, there were no ACTH concentrations above 1,000 pg/mL. In 92.3% of the cases, potassium levels were low, with a mean of 2.3 mEq/L (SD 0.7), (median 2.1 IQR 0.95). Hypokalemia was difficult to manage in most of the patients: 50% of patients required oral and venous potassium reposition, 14% had oral reposition only, 14% did not have hypokalemia and 21% of patients did not have available data about reposition. This finding was not associated with complications such as arrhythmias, neuromuscular compromise, or sudden death.

**Table 3:** Biochemical profile

Case	Diagnosis	Cortisol 8 am	Cortisol LLDST ug/dL	Cortisol HDDST ug/dL	UFC ug/24 h!	ACTH pg/mL	Lowest potassium mEq/lt
1	Occult	68	NR	NR	3350	265	1.8
2	Metastatic medullary thyroid carcinoma	27.4	24	NR	2600	NR	2.6
3	Well-differentiated pancreatic neuroendocrine tumor	21.7	7.7	7.2*	NA	31.8	3
4	Lung carcinoid	21 24.5†	22.2 4.5†	18.42 -	NR -	NR 401.4†	NA
5	Small-cell lung carcinoma	33.66	22.6	30.61	NR	32.5	1.3
6	Lung carcinoid with liver and bone metastases.	NR	28.8	NR	3994	93.7	2.2
7	Probable small-cell lung carcinoma	NR	44.6	> 120	6963	115.9	1.7
8	Grade 3 pancreatic neuroendocrine carcinoma with liver metastasis	NR	45.9	48.5	6487	10.2	2.1
9	Lung carcinoid	34.2	32.5	28.9	283	88.3	2.8
10	Pheochromocytoma	19.4	23.8	2.1*	98	25.8	4.03
11	Poorly-differentiated lung neuroendocrine carcinoma with liver metastasis	-	58.9	-	7530	79.9	1.8
12	Grade 2 pancreatic neuroendocrine carcinoma with liver metastasis	58.7	NR	63.7	196.3	135.6	1.9
13	Metastatic thymoma	21	49	29	1478	144	2.7
14	Small-cell lung carcinoma	60	60	-	253	765	2.1

ACTH: Adrenocorticotropic hormone; UFC: 24-hour urinary free cortisol; NA: not available; NR: not realized; LLDST: low-dose dexamethasone suppression test (LDDST); HDDST: high-dose dexamethasone suppression test.

! Reference levels differ according to laboratory (HSVF 58-243 ug/24 h, HPTU 50-190 ug/24 h)

† At the time of recurrence.

* Suppression of plasma cortisol after HDDST by 50 percent of basal value.

### Localization studies

In the imaging studies, the tumor causing the ECS was found in 92.8% of the 14 patients. Of the patients, 42% had octreoscan, but it was only useful in 7.1%. The majority of cases had a diagnosis with CT (57%) and MRI (28.5%); 14.2% had 18 FDG-PET/CT and 7.1% were diagnosed by an abdominal ultrasound. In 45.4% of the patients, adrenal hyperplasia was found in images, none of which showed nodular hyperplasia. Sinus petrous catheterism was done in 4 (26.6%) cases and in all cases were negative for pituitary source.

### Management

Table 4 shows the treatments received by the patients and their outcomes. Surgical resection of the primary tumor was performed in 50% of the patients. There were no available measurements of urinary free cortisol post-resection to determine if the excessive secretion improved. In the patient with an occult tumor (Case No 1) and the medullary thyroid cancer case (Case No 2), bilateral adrenalectomy solved the hypercortisolism. Case No 2 also received management with long-acting somatostatin analogues for chronic diarrhea associated with her cancer, without improvement in ECS. Ten (71.4%) of the 14 patients received inhibitors of steroidogenesis (ketoconazole) and four (28.5%) received somatostatin analogues (Octreotide LAR). Four patients required bilateral adrenalectomy (21.4%) and the pheochromocytoma case needed a right adrenalectomy. Four (28.5%) of the 14 patients required other treatments: chemotherapy with carboplatin, etoposide and dexamethasone (n = 2, 14.3%); liver chemoembolization with doxorubicin (n = 1, 7.1%) and topotecan and holoencefalic radiotherapy (n = 1, 7.1%).

**Table 4:** Treatment and outcome

Case	Age	Sex	Diagnosis	Surgical management	Medical management	Other therapies	Outcome, time after ECS diagnosis	Control of hypercortisolism
1	32	M	Occult	Bilateral adrenalectomy	Ketoconazol* 800 mg/d	-	Occult ( 1 month)	Yes
2	28	F	Metastatic medullary thyroid carcinoma	Thyroidectomy + bilateral adrenalectomy+ thrombectomy of inferior vena cava .	Somatostatin analogue		Death (3 months), disease progression	Yes
3	46	F	Well-differentiated pancreatic neuroendocrine tumor	Distal pancreatectomy + open bilateral adrenalectomy	-	-	Alive (132 months)	Yes
4	31	F	Lung carcinoid	Resection of lung tumor (1986-2006)	-	-	Alive (96 months)	Yes
5	72	F	Small-cell lung carcinoma	Bilateral adrenalectomy	-	Carboplatin, etoposide, dexamethasone	Death (febrile neutropenia – septic shock, 3 months)	Yes
6	62	M	Lung carcinoid with liver and bone metastases.	Lung lobectomy plus right bronchoplasty in 2004	Ketoconazol 600 mg/d Octreotide LAR 30 mg/m	Liver trans-arterial chemo-embolization with doxorubicin	Death 29 months (pneumonia)	No
7	68	M	Probable small-cell lung carcinoma	-	Ketoconazol 400 mg/d	-	Death (respiratory failure/sepsis) 2 months	No
8	77	M	Grade 3 pancreatic neuroendocrine carcinoma with liver metastasis	-	Ketoconazol 800 mg/d Octreotide SC 0.3 mg sc q/d	-	Death, 15 months, disease progression	No
9	70	F	Lung carcinoid	Segmental lobectomy of medial lobe	Ketoconazol 600 mg/d*	-	Alive (27 months)	Yes
10	59	F	Pheochromocytoma	Laparoscopic right adrenalectomy	-	-	Alive (31 months)	Yes
11	64	F	Poorly-differentiated lung neuroendocrine carcinoma with liver metastasis	-	-	Carboplatin, etoposide, dexamethasone,	Death (septic shock, 1 month)	No
12	68	F	Grade 2 pancreatic neuroendocrine carcinoma with liver metastasis	Distal pancreatectomy	Ketoconazol 800 mg/d Octreotide LAR 30 mg/m	-	Death 27 months (disease progression)	Yes
13	35	M	Metastatic thymoma	-	Ketoconazol 600 mg/d	-	Alive (33 months)	Yes
14	50	M	Small-cell lung carcinoma	-	Ketoconazol 800 mg/d	Topotecan Cranial Radiotherapy	Death 22 days (disease progression)	Yes

* Prior to tumor resection.

### Outcomes

**Figure 1:**
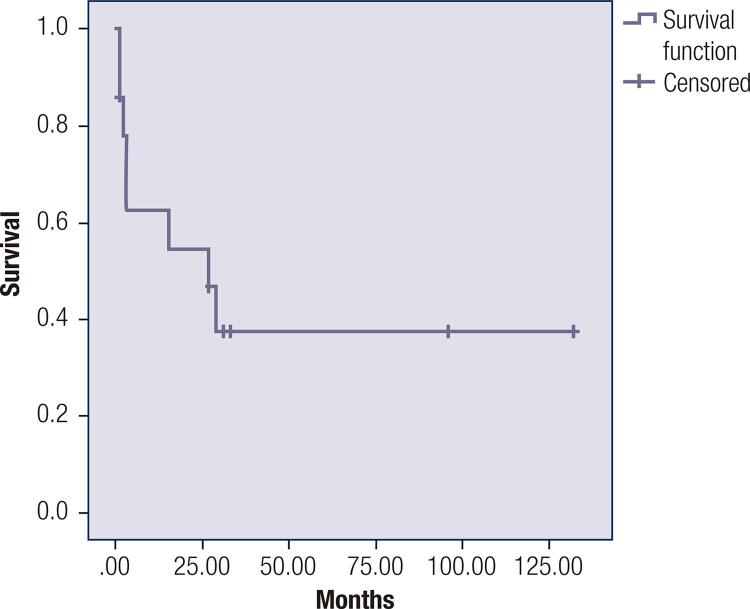
Overall survival of ectopic Cushing syndrome. Mortality was 57.1%, median survival was 27 months (IC 95%: 1.1-52).

There were eight deaths (57.1%); all of these patients had advanced disease and died due to tumor progression or complications related to chemotherapy (14.2%). Median survival was 27 months (95% CI 1.135- 52.8) ( Figure 1 ). Patients with resectable tumors had a longer survival compared with patients with unresectable tumors (resectable: median not achieved, unresectable median 3 months [95% CI 0 -13.4, *p* = 0.013, Figure 2 ]). Unresectable tumors had a high mortality (85%).

Bilateral adrenalectomy was performed in 4 patients (21.4%). Two (MTC and SCLC) had unresectable primary tumors (50%), one case had an occult ECS and the other case had a pNET. Two cases died less than three months after adrenalectomy and the occult ECS was lost from follow-up. A patient with pNET had a resectable tumor and after adrenalectomy had a survival of 124 months.

## DISCUSSION

This is the first report on the challenges that patients with ECS may face in a tropical country. Similar to reports derived from other latitudes, patients with ECS are overwhelmingly affected by proximal muscle weakness, severe osteoporosis, hypokalemia, diabetes mellitus, hypertension and cardiovascular disease. However, patients from tropical countries may face a different and more challenging spectrum of opportunistic infectious diseases. For instance, our patients faced malaria, tuberculosis, and other tropical infections. However, this was not reported in a previous (16). In fact, endemic infectious diseases of various etiologies have been previously described in patients with ECS. Examples of these conditions are parasitic infestations such as strongyloidiasis, systemic mycosis such as paracoccidioidomicosis, and viral disease. In the context of ECS, the manifestations of these infections could be very severe and their clinical manifestations may be atypical and frequently overlapping, making their identification challenging. For instance, tuberculosis may be confused with either cytomegalovirus or pneumocystis infections. Patients with ECS may subsequently require multiple tests to confirm or rule out the broad spectrum of endemic conditions, their diagnosis may be delayed leading to increased mortality, and expenses are quite high. Clinical experience is very important. It is important to emphasize that common infections, such as community acquired pneumonia, are also prevalent and that treatment plans should be developed accordingly.

Our report suggests that ECS is slightly more common in women than men. This is similar to what was previously reported by the MD Anderson Cancer Center ( 12 ), but different from a multicentric Italian study that did not find a difference in incidence based on gender ( 17 ).

Manifestations related to proteolysis of supportive proteins such as severe osteoporosis, proximal muscle weakness and skin fragility as well as metabolic abnormalities (primarily hypokalemia) were the most prevalent. These manifestations clearly indicate that patients faced glucocorticoid toxicity at maximal expression. For instance, the saturation of 11-β-hydroxysteroid dehydrogenase type 1 by cortisol increases the availability of cortisol to interact with the mineralocorticoid receptor and facilitates the development of hypokalemia ( 18 ). Patients required large amounts of both oral and intravenous potassium replacement therapy along with spironolactone while waiting for medications such as ketoconazole to inhibit the excessive secretion of cortisol. Conversely, patients with milder forms of Cushing syndrome present less often with hypokalemia (adrenal adenomas, Cushing disease). Only 36% of our patients had hyperpigmentation; in fact, most of our patients presented with ACTH values < 150. Nevertheless, the production of ACTH is proportional to the tumor burden and patients with extensive metastases will likely be more hyperpigmented.

The biochemical diagnosis of Cushing syndrome was mainly made by elevated urinary free cortisol, and the absence of cortisol suppression using the low-dose dexamethasone suppression test. Of the biochemical tests, the most impressive results were obtained from the 24-hour urine collection of cortisol. More than half of the patients had urinary free cortisol concentrations at least 10 times above the reference value; our experience with salivary cortisol is limited as it is not widely available in the region. As expected, most of our patients had neuroendocrine tumors. Of the ACTH-producing tumors, 57.1% were intrathoracic, similar to the rates reported in other series ( 19 , 20 ), which were followed by intra-abdominal tumors in prevalence. In our series, 93% of our patients had tumors that were well-characterized by conventional imaging (CT /MRI). Some small studies with octreoscan suggest a median sensitivity of 84% (57%-93%) for the identification of lesions ( 21 ). In our series, the octreotide scan did not provide clinicians additional information from that provided by CT/MRI and, subsequently, the clinical approach did not change. The octreoscan was normal in the only patient with ECS of an unknown origin in our series. Sinus petrous catheterism is not available routinely in our country. Our center in Medellin, performs sinus catheterism with desmopressin given the unavailability of CRH in our country.

The localization of an ACTH-secreting tumor is challenging. In the series published by a Mexican group, 50% of patients had an occult ECS ( 22 ). In our series, despite the judicious evaluation, in one (7.14%) out of 14 cases the cause of ECS remained occult. This percentage is slightly lower than the rate of 12-16% reported by others ( 18 , 19 ) and is similar to the rate reported by MD Anderson (9.3%) and Brazil experience (8%) ( 16 ). DOTATATE PET/CT has shown to be a very sensitive test to characterize patients with several types of neuroendocrine tumors. Its ECS value remains to be determined. Patients with poorly differentiated tumors may not express somatostatin receptor 2 ( 23 , 24 ). Furthermore, extensive clinical experience suggests that most patients with ECS treated with somatostatin analogues do not respond to this therapy ( 24 , 25 ). Access to this sophisticated and expensive technology is limited in our region.

Three (21.4%) patients had episodes of deep venous thrombosis, predisposing them to pulmonary embolism, which is an important cause of morbidity and mortality in these patients ( 23 , 24 ) requiring strict monitoring. Low-molecular weight heparin prophylaxis associated with elastic compression of the lower extremities is suggested in all patients hospitalized with ECS. Four (28.5%) patients died during disease progression.

The optimal treatment of ECS is the surgical resection of the primary tumor. This was achieved in 7 (50%) patients. Despite the surgical resection of the primary tumor, three patients developed (21.4%) metastatic disease with exacerbation of Cushing’s syndrome. The curative resection rate was 28.5%, contrasting with the 12% rate reported in the Mayo Clinic series ( 19 ), this series was done long time ago ( 19 years). There have been several improvements in health care, for example, surgical technique and post-operative care is better now and surgical treatments are more aggressive. Also, they reported a higher percentage of primary unknown tumor than our cohort which limits the role of surgical treatments. Primary resection is one of the main prognostic factors in patients with ECS ( 17 ). For this reason, the primary tumor must be found and resected by an experienced center.

When the resection of the primary tumor is not possible, bilateral adrenalectomy is an option. In our series, bilateral adrenalectomy for the control of hypercortisolism was offered to 21.4% of cases to allow the control of hypercortisolism and associated diseases in these patients. The ideal patients for bilateral adrenalectomy are those with an acceptable life span and a good performance status ( 26 ). For example, in our series, patients with pNET and a good performance status benefited from bilateral adrenalectomy. Patients with SLCL had very aggressive disease with a life span no longer than 6 to 12 months. For these patients, treatment with medications that inhibit adrenal steroidogenesis is preferable. In most countries, metyrapone is not available. Our patients were treated with ketoconazole. However, responses were limited.

The tumors with the worst prognosis were intrathoracic lesions; 6 (75%) of the 8 cases with tumors located in the thorax series died. Survival amongst series varies: MD Anderson (33.2 months), Italy (60 months), NIH (26 months), and ours (27 months). These differences in survivorship confirm that the nature of the causes of ECS are heterogeneous ( 12 , 21 ).

Nevertheless, one of the most important factors for the improvement of survival in our series is the possibility of resecting the tumor, improving cortisol hypersecretion and curing ECS. Several other factors that affect mortality include age, presence of metastases, tumor type, urinary cortisol and ACTH, potassium levels and diabetes ( 17 ).

Our study has limitations associated with the retrospective design, for example some of the symptoms and comorbidities may not have been captured accurately. And sample size that did not allow us to perform a multivariable analysis. Nevertheless, it describes the challenges that patients with ECS and providers may face in tropical regions.

In conclusion, ECS is a rare endocrine disease associated with a high rate of complications and mortality. This report may serve as a reference for studies at the national or regional level and may lead to a more careful identification of co-morbidities as well as a more cost-effective approach.

Our series shows the necessity in developing countries of an adequate biochemical evaluation, localization studies, and an expert’s center with endocrinologists and surgeons for ECS treatment. This study may lead to the formation of groups of excellence for the management of CS and impact the survival and quality of life of those affected with this uncommon disease.
